# An Observed Regime Shift in the Formation of Warm Core Rings from the Gulf Stream

**DOI:** 10.1038/s41598-019-48661-9

**Published:** 2019-08-23

**Authors:** Avijit Gangopadhyay, Glen Gawarkiewicz, E. Nishchitha S. Silva, M. Monim, Jenifer Clark

**Affiliations:** 1 School for Marine Science and Technology, University of Massachusetts Dartmouth, MA, 02747 USA; 2 Woods Hole Oceanographic Institution, Woods Hole, MA, 02543 USA; 3 RPS Group, 55 Village Square Drive, South Kingstown, RI 02879 USA; 4 Jenifer Clark’s Gulfstream, 3160 Lacrosse Court, Dunkirk, MD 20754 USA

**Keywords:** Projection and prediction, Physical oceanography

## Abstract

We present observational evidence that a significant regime change occurred around the year 2000 in the formation of Warm Core Rings (WCRs) from the Gulf Stream (GS) between 75° and 55°W. The dataset for this study is a set of synoptic oceanographic charts available over the thirty-eight-year period of 1980–2017. The upward regime change shows an increase to 33 WCRs per year during 2000–2017 from an average of 18 WCRs during 1980 to 1999. A seasonal analysis confirms May-June-July as the peak time for WCR births in agreement with earlier studies. The westernmost region (75°-70°W) is least ring-productive, while the region from 65°W to 60°W is most productive. This regime shift around 2000 is detected in WCR formation for all of the four 5-degree wide sub-regions and the whole region (75°-55°W). This might be related to a reduction of the deformation radius for ring formation, allowing unstable meanders to shed more frequent rings in recent years. A number of possible factors resulting in such a regime shift related to the possible changes in reduced gravity, instability, transport of the GS, large-scale changes in the wind system and atmospheric fluxes are outlined, which suggest new research directions. The increase in WCRs has likely had an impact on the marine ecosystem since 2000, a topic worthy for future studies.

## Introduction

Continental shelf waters along the mid-Atlantic and northeastern US have been rapidly changing over the last ten years. Recent observational studies indicate that extreme warming conditions are occurring more frequently in the water masses from the Middle Atlantic Bight (MAB) to the Gulf of Maine/Georges Bank (GOM/GB), along and across the Shelf break Front (SBF), in the slope waters and on the Labrador Shelf all the way into the Arctic^1–5^. Changes have been documented in circulation and water masses, ecosystem response, fisheries abundance, fish recruitment and seasonal migration^6,7^.

Pershing *et al*.^5^ stated that during the last decade, sea surface temperature in the GOM increased at a rate faster than 99% of the global oceans. They attributed such changes to factors such as the northward excursion of the GS and changes in the Atlantic Multi-decadal Oscillation and Pacific Decadal Oscillation. These authors also maintained that such changes might have caused the collapse of the cod fishery in New England waters^2,8,9^.

While observational evidence for change is growing, there are competing theories on how these changes are brought about. During 2012, winter and spring shelf water temperatures were the warmest on record^2,10^. This was attributed to decreased heat loss by the ocean during winter due to a northward shift of the atmospheric Jet Stream, and consequent warming of shelf waters^11^.

One of the major drivers of the changes in the shelf and slope waters off the US northeast coast is thought to be the latitudinal excursions of the GS bringing warm waters into the slope sea in the form of multiple Warm Core Rings (WCR) and streamers/shingles from the GS. Determining the impact of the WCRs on the shelf-slope exchange and thus on the water masses on the shelf^12–15^ is one of the priority areas of the Ocean Observatories Initiative science plan for the Pioneer Array^15^ and is presently a major area of active research^11,16,17^. Their frequent occurrence and impact on the physical, chemical and biological oceanography of the Slope Sea region have been documented in the past through field observations^18–20^, satellite imagery^21–24^ and theoretical models^25–28^. However, a systematic study of WCR formation and distribution is necessary to understand the impact of the rings on the underlying ecosystem and its habitats.

Previous climatological studies were limited by the number of years of data availability. For example, a number of studies^23,24,29^ used different 5-year charts to characterize WCR formation and propagation statistics. A consistent 38-year-long (1980–2017) dataset documenting the occurrence and pathways of the WCRs in the GS region (75°W-55°W) on a semi-weekly basis has been compiled for this study^30,31^. First results from a comprehensive analysis based on a rigorous census developed from this 38-year-long database are presented here. One of the primary objectives is to determine the spatial variation of the seasonal and inter-annual variability of WCR formation along the GS path from 75°W to 55°W. In doing so, we uncovered a distinct regime-shift in the number of WCRs formed after 2000. This paper focuses on this observed regime-shift and discusses a number of probable dynamical factors behind the regime-shift that may suggest new directions of research.

## Results

**Figure 1:**
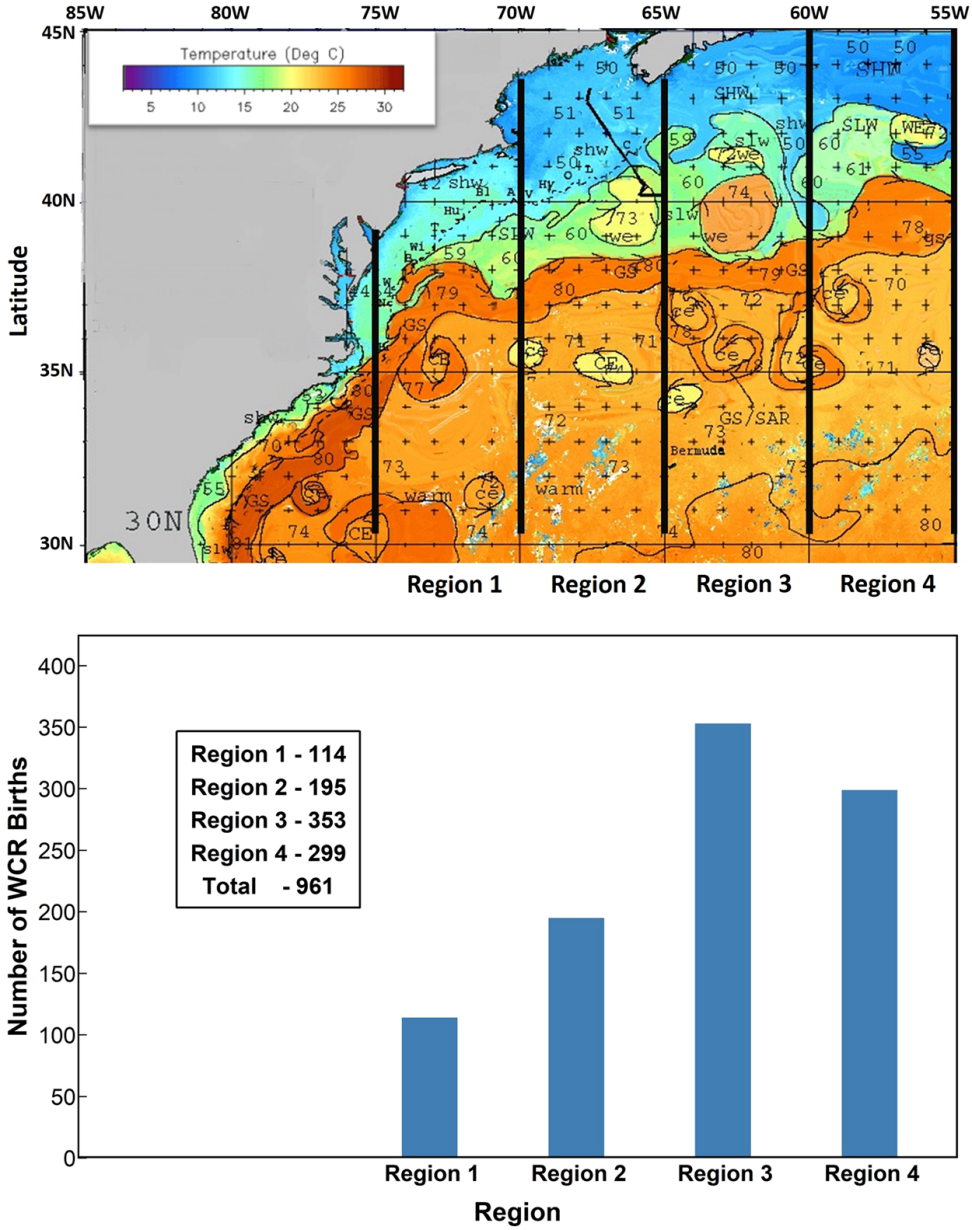
(**a**) An example of a GS Chart from the analysis of Jenifer Clark. The four sub-regions of 5-degree bins are shown as separated by thick black lines. (**b**) Region-wide distribution of WCR formation during 38 years of study (1980–2017). Region 1: 75°-70°W; Region 2: 70°-65°W; Region 3: 65°-60°W; Region 4: 60°-55°W.

Our main data is a set of charts prepared by one of the co-authors (Jenifer Clark) from 1980 through 2017. An example Chart with annotations of features (GS, WCR, CCR, shelf slope front, other eddies and features) is shown in Fig. 1a. NOAA and the Bedford Institute of Oceanography (BIO) used these charts from 1980 to 2004 for extracting the GS and its eddy locations, sizes and migration. We reprocessed all the charts from 1980 to 2017 using GIS to establish a comprehensive, consistent and accurate database (see Methodology for details). A robust census for WCR births was developed for the full region (75°W-55°W) and for four sub-regions (Region 1: 75°W-70°W; Region 2: 70°W-65°W; Region 3: 65°W-60°W and Region 4: 60°W-55°W) (See Fig. 1a). During the 38-year study period, out of a total of 961 WCRs formed, Region 1 had only about 12% (114) of the total and Region 2 gave birth to about 20% (195) of the Rings (Fig. 1b). The more productive regions to the east had 37% (353) and 31% (299) for Regions 3 and 4 respectively. The New England Seamount Chain (NESC) underlies the Gulf Stream in the northeastern part of region 2 and in the southwestern part of region 3, possibly accentuating large-scale GS meandering enhancing the WCR formations in regions 3 and 4^32^.

### Seasonal-to-inter-annual variability

**Figure 2:**
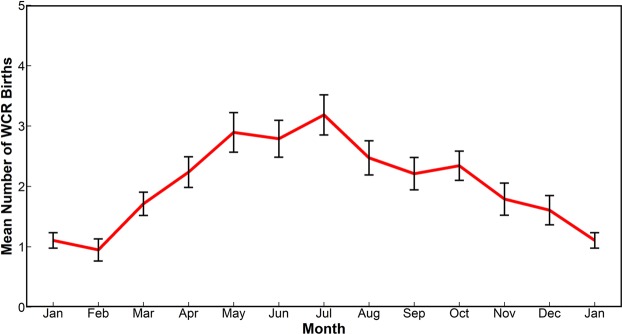
Seasonal Cycle of WCR formation over the whole region between 75°W and 55°W. The vertical bars denote the standard error of mean for each month.

On a seasonal scale, WCR formation peaks in late spring/early summer (May-June-July) while the wintertime (January-February) has fewer rings forming (Fig. 2). The summertime peak is also present in each of the four different sub-regions. Previous statistical studies on WCRs have also indicated that the ring production by the GS system peaks during the summer months^23,24^.

The WCR formation process has been linked with GS instability processes, which convert the available potential energy to the eddy kinetic energy (EKE)^33–35^. Zhai *et al*.^36^ analyzed satellite altimeter data and found that in the GS region (73°W-44°W), EKE peaks in summer while the ocean is most baroclinically unstable during the winter. A recent numerical modeling study^37^ found that in the GS region (75°W-55°W) EKE has a dominant peak in May and a secondary peak in September near the surface. A similar correlation between surface EKE and the baroclinic instability was observed in the North Pacific^38^ and the southern Indian Ocean^39^. In these cases, a theoretical model was used to show that the lag of a couple of months corresponds to the length of time for unstable waves to grow in the respective regions.

**Figure 3:**
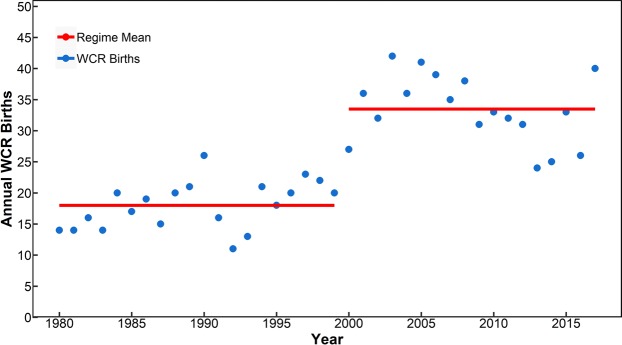
Interannual Variability of the WCR formation between 1980 and 2017. The regime shift (denoted by the split in the red solid line) is significant at the turn of the century.

The observed annual birth of the WCRs for the whole time-period (1980–2017) is presented in Fig. 3. From a sample size of 961 WCRs, there is significant inter-annual variability in the number of WCRs formed in individual years, with a maximum occurrence of 42 in 2003 (followed by 41 in 2005 and in 2017), and a minimum occurrence of 11 WCRs in 1992. The number of WCRs in the slope sea between 75° and 55°W has significantly increased over the 38-year period (1980–2017). The inter-annual variability consists of short periods of increasing and decreasing rates of ring formation; the maximum rate was seen between 1993 and 2005, when about 2 additional rings were born every year, followed by a decreasing rate between 2005 and 2012. A more recent increasing rate (2012–2015) has been discussed briefly by Gawarkiewicz *et al*.^40^ in relation to recent warming of the Gulf of Maine and Northeast Shelf ecosystem.

### Regime shift around the year 2000

**Figure 4:**
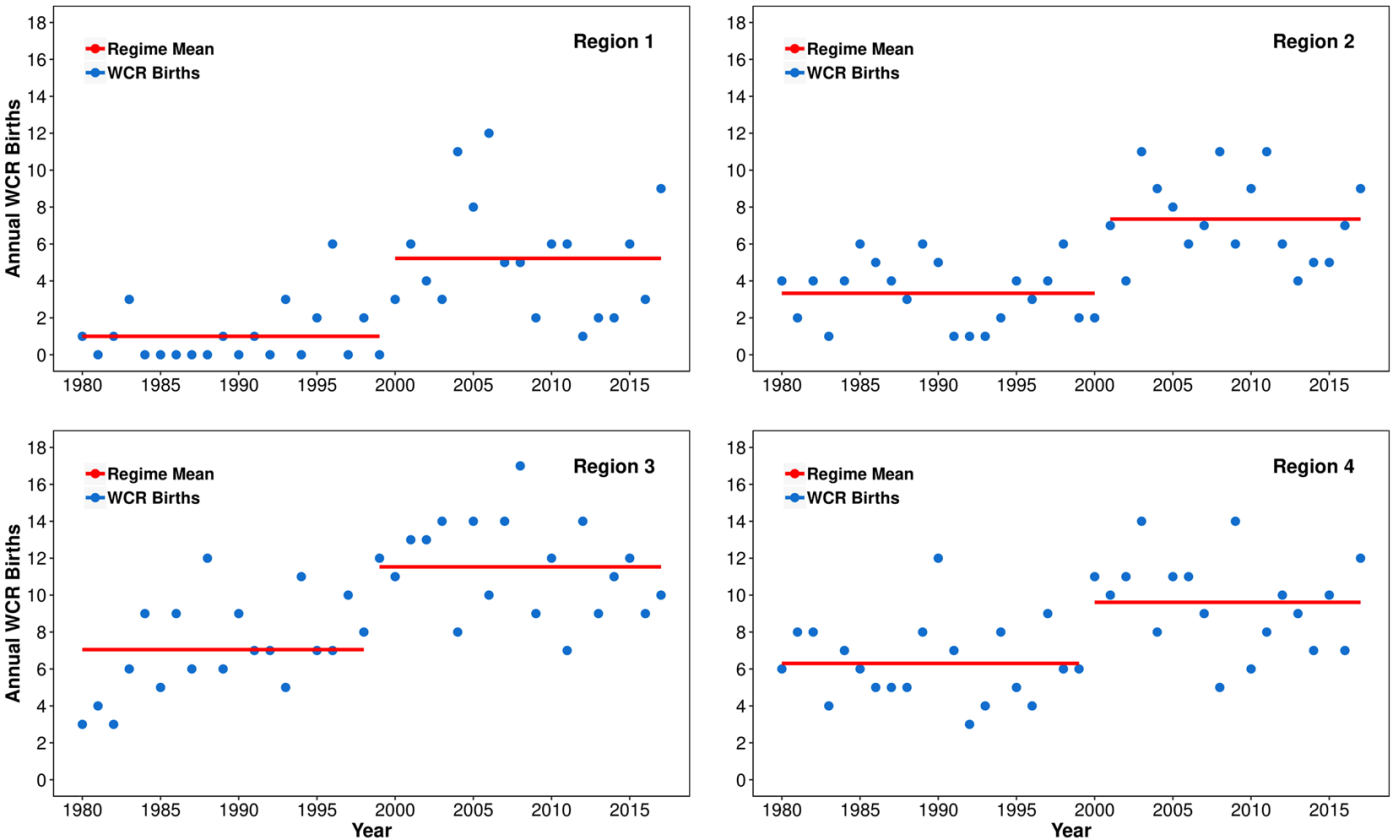
Interannual Variability of WCR formation in different sub-regions–Region 1: 75°-70°W; Region 2: 70°-65°W; Region 3: 65°-60°W; Region 4: 60°-55°W. Significant regime changes were detected between 1998 and 2000 for each region. See Table 1 for exact shift years.

Given the pattern of ring formation appearing in Fig. 3, the possibility of an abrupt change in the pattern opposed to a gradual increase is worth examining. Regime shifts are a common feature of many geophysical systems^41–44^ and it is unclear a priori whether abrupt or gradual change should be expected in ring formation for the Gulf Stream system. In this study, a sequential-t test-based regime shift detection algorithm^45–47^ was used to identify the regimes evident in the WCR birth time-series shown in Figs 3 and 4. The method of detecting regime shift is described in the Methodology section.

It is evident that the WCR formation process has gone through a regime shift around 2000. Note that the period 1980–1999 produced a total of 360 rings (annual average of 18), while the period (2000–2017) produced a total of 601 rings (annual average of 33). Figure 4 presents the regime-shift analysis results for each of the four sub-regions. The summary statistics of the regime-shift analysis are presented in Table 1. All four sub-regions show significant regime change between 1998–2000. These results were also supported by the Change-point analysis in R^48^, the Change-point detection in Matlab^49,50^ and by an independent Markov Regime Switch model^51^, in that these methods also detected the regime-shift for the whole area in year 2000 and for the sub-regions between 1998–2000 (see Methodology for details on these different models).

**Table 1:** Summary of statistics for the Regime Shift analysis for all regions.

Regime Character		Region 1 (75–70 W)	Region 2 (70–65 W)	Region 3 (65–60 W)	Region 4 (60–55 W)	Total WCR (75–55 W)
Regime #1	Period	1980–1999	1980–2000	1980–1998	1980–1999	1980–1999
	Mean	1.00	3.33	7.05	6.30	18.00
	L	20	21	19	20	20
Regime Shift	Shift	Upward	Upward	Upward	Upward	Upward
	RSI	8.47	9.24	10.84	7.34	15.25
	p-value	2.81E-05	2.17E-06	3.95E-06	1.13E-04	5.79E-11
Regime #2	Period	2000–2017	2001–2017	1999–2017	2000–2017	2000–2017
	Mean	5.22	7.35	11.53	9.61	33.39
	L	18	17	19	18	18

The time-series in Fig. 3 also shows an overall increasing trend that could fit a linear model. Significant p-values were obtained for both linear and regime-shift models. However, the residual variance (the variance of the residual between the observations and the model fit)^52^ was larger (36.91) for the linear model compared than for the regime-shift model (21.47). Furthermore, the regime-shift model explains 75% of the variance compared to only 56% by the linear trend. Thus we conclude that the regime-shift model renders an appropriate and robust explanation of the behavior of the WCR formation during this 38-year period.

## Discussion

Three different factors are important to consider for investigating possible reasons behind such a regime shift of the WCR formation: (i) decreasing reduced gravity, (ii) internal GS dynamics and (iii) atmospheric forcing.

A typical ring formation event after the GS leaves the coast at Cape Hatteras happens when the radius of deformation is comparable to the meandering length scale^53–55^. Dynamically, this occurs when the centrifugal force is balanced by the Coriolis force for the fluid parcels following the crest of a meander, which then becomes a closed vortex, or WCR. The internal radius of deformation (R_d_) is generally given by $(\frac{\sqrt{g^{\prime} H}}{f})$, where *g*′ is the reduced gravity, H is the water depth, and f is the Coriolis parameter.

Therefore, a reduction of *g*′ might lead to a smaller R_d_ and increased WCR formation. The observed warming in the slope waters^4,40,56^ in the past decade might have reduced the density difference between the slope water and the GS. So, the recent warming in the slope water might have contributed to the increasing number of WCRs in the later regime after 2000. Additionally, while atmospheric forcing might have led to the initial warming of the slope sea^10^ the latter mechanism of decreasing reduced gravity has a positive feedback by producing more WCRs giving rise to even warmer and saltier slope water.

It is also reasonable to postulate that the WCR formation is driven by the instabilities (both barotropic and baroclinic)^53,57^ generated in the GS through its interaction with the slope and Sargasso waters, with the Deep Western Boundary Current (DWBC) and with the NESC. However, the instabilities take time to grow and thus a lag between the transport of the GS at Hatteras and the WCR formation in the regions downstream might be expected as discussed in Section 2.1. In this context, the multi-year (1992–2016 and beyond) transport data for the GS, Sargasso and Slope waters available from the Oleander group^58–60^ would be very useful. A thorough instability-based analysis relating the OMV Oleander transport (of the GS, Sargasso and Slope waters), westward movement of the destabilization point of the GS^57^, the DWBC strength and proximity to NESC with the number of WCRs will be forthcoming.

Further to the east, an altimetric data analysis^61^ showed that the GS path between 65°W and 55°W has progressively moved southward during 1993–2013. The overall increasing trend of the WCR formation over almost four decades also coincided with a recently reported southward shift east of 65°W and slowing of the GS transport^62,63^ during 1993–2016. Evidently such a southward excursion of the GS system at its eastern end would allow for more WCR birth in the 65°-55°W region and might have resulted in increased ring formation during the last seventeen years. Note that both sub-regions (65°-60°W and 60°-55°W) are the major contributors to the total ring formation numbers due to the stream’s large-amplitude meandering behavior as it crosses the NESC^32,64^. Thus, a slight additional southward displacement might enhance ring formation in this region even further due to flow interactions with the Sea Mounts.

With regards to atmospheric forcing, the North Atlantic Oscillation (NAO) has been linked to the formation of the WCRs through the GS EKE in the past^16,35,65^. While the WCRs were inversely lag-correlated with the NAO winter Index during 1978–1999^16^, such a relationship with the NAO was not found during 2000–2016. It is uncertain at this time how the interannual variability of the NAO-induced winds affect the GS EKE to provide for the baroclinic instability that would be necessary to produce a large number of WCRs during the past two recent decades.

One other obvious suspect for the causes of the regime shift is the wind-stress curl over the subtropical North Atlantic that generates the westward propagating Rossby waves to generate the western boundary current^66–69^. Recently, the decadal shifts of the Kuroshio Extension (KE) have been shown to be associated with a weak (strong) transport and unstable (stable) meandering configuration^70^. These opposing phases were linked to the basin-wide wind-stress curl forced negative (positive) Sea Surface Height (SSH) anomalies propagating west in the form of Rossby waves during negative (positive) phases of the North Pacific Gyre Oscillation. Furthermore, Yang *et al*.^71^ recently showed that the strong and stable state of the KE is also associated with a strong southern recirculation gyre. Future studies are needed to investigate the possibility of a weakening southern recirculation gyre during the past two decades that could add to the increasingly unstable state of the GS. Such investigations should also reconcile with recent observations of westward movement of the destabilization point of the GS^57^.

Such a high number of WCRs in the slope water might have impacted the ecosystem of the GOM/GB and MAB by making them even warmer and saltier in the first two decades of the twenty-first century. This is clearly evident during recent specific Ring intrusion events, for example during January 2017 south of New England when Gulf Stream flounder were caught in Rhode Island Sound in addition to juvenile Black Sea Bass^40^. However, it is also likely that the increasing frequency of warm core ring encounters with the continental shelf will contribute to increased warming of the continental shelf. This in turn is likely to increase the rate at which the geographical centroid of marine species moves to the north^72^.

## Conclusions

We present observational evidence that the number of WCRs formed from the GS has undergone a significant regime-shift at around the year 2000. The average number of WCR formations has increased to 33 per year during 2000–2017 from an average of 18 per year during 1980–1999. We hypothesize that the increase of the number of WCRs in recent years could be related to increased instability due to several factors, such as (i) decreasing reduced gravity between the slope and the GS due to warming of the slope (via atmospheric forcing), (ii) internal dynamics of the GS system (including transport, latitudinal movement, and interactions with DWBC and NESC), and (iii) changes in the large-scale atmospheric forcing, or a combination of these factors. Further detailed simulations and energetics analysis will be necessary to quantify these relationships and identify the dynamics behind the increased number of WCRs since 2000.

## Methodology

### Data

The primary dataset is a set of charts prepared by one of the co-authors, Jenifer Clark (JC). An example is shown in Fig. 1a. This collection of charts of the GS and surrounding waters has been annotated with satellite data indicating temperature. Using infra-red (IR) imagery, satellite altimetry data, and surface *in-situ* temperature data, oceanographic analyses were produced for this region in the form of 2–3 day composite charts in a consistent manner. These charts show the location, extent and temperature signature of currents (GS, shelf-slope front), warm and cold-core rings (WCRs and CCRs), other eddies, shingles, intrusions and other water mass boundaries in the Gulf of Maine, over Georges Bank and in the Middle Atlantic Bight.

These charts have been used in the past by various researchers for different purposes. Some studies^23,24,29^ have used these over different 5-year periods in the 1980s to develop a WCR climatology and related statistics. These charts were used for the first synoptic prediction of cold-core-ring propagation and their acoustic signatures for the US Navy^53^. Such charts were also used for interannual variability studies^16^ and for IOOS-related operational forecasting^73,74^.

The basis data source was individual IR temperature images from the NOAA polar orbiting satellites (NOAA-5 in the early 1980s to NOAA-18 recently) at 6–12 hourly intervals. These images were captured by the Advanced Very High Resolution Radiometer (AVHRR) and AVHRR2 instruments, both of which had a resolution of 1.1 km over the last four decades. Each individual image has a different lookup table (or colormap) for temperature that resolves 256 distinct sets of intensity, hue and saturation of color within the available and retrievable IR signal range. This allows for accurate identification of the small-scale features in each image. The analyst locates all of the small scale features in each individual satellite SST image within a three-day period. The locations and boundaries of the features (GS, WCR, CCR and other smaller scale entities) are remapped onto a 3-day composite image for that period. The 3-day composite image has a fixed and broad (5–30 °C) range of temperature with similar 256-set indexing, which by itself could not resolve the features. Note that individual images with high-resolution within a narrower band of temperature range also have clouds, which are eliminated (or at least minimized) during the process of generating the 3-day composites. The 3-day composite helps to visualize the whole GS and its rings in a broader region (like Fig. 1a); while the individual images help resolve the features at a very high resolution. The 3-day composite images are regularly produced by NOAA and/or the Johns Hopkins University Applied Physics Lab (fermi) group (see http://fermi.jhuapl.edu for more details). Thus, the JC Charts, which uses this basis data source, is the most continuous and consistent data set to extract the WCRs, the GS and the CCRs over the whole period of analysis (1980–2017) at a constant resolution of 1.1 km^30,31^.

The process of creating the WCR census time-series can be summarized as follows. First, the JC Charts are available 2–3 times a week from 1980–2017. Thus, we used approximately 5000 Charts for the 38 years of analysis. All of these charts were reanalyzed between 75° and 55°W using QGIS 2.18.16^75^ and georeferenced on a WGS84 coordinate system^76^. The analyst goes through each chart and follows a set of rules (birth, continuity, death) to identify each WCR^30^ and tabulates the ring parameters. A new ring formation is documented in the following situations: (i) a typical GS crest forming a closed anticyclonic vortex and detaches from the stream in the slope water; (ii) an anticyclonic eddy forms off of another large anticyclonic eddy in the slope water; (iii) an anticyclonic eddy further away from the stream coming into the domain through Region 4^30^. Note that any anticyclonic eddy that existed for less than 7 days was not counted in the census.

Thirty-eight years of WCR census yielded a total of 961 WCRs and their birth, death, size and age information were documented and are available on request. In addition, we also have access to a database from Roger Pettipas of  BIO who documented the ring center location, and size at birth on each analysis day, generally twice a week from the same set of JC Charts (also called the NOAA Charts) during the period 1980–2004. A validation was carried out^30,31^ using the BIO data, an earlier study^16^ and this new Census to eliminate the possibility of any analyst error. A similar and comprehensive Census development for the CCRs of the GS system using the GIS framework is underway.

### Regime shift analysis

A sequential regime shift detection algorithm^45–47^ was used to identify the regimes evident in the WCR birth time-series shown in Fig. 1 for the whole and all four sub-regions. The algorithm detects the regime shifts in the mean and the variance. Briefly, the method includes applying the student’s T-Test sequentially to a time-series when data is arriving continuously. With the arrival of a new observation to its time-series, a check is performed to determine whether the deviation of the current mean, $\overline{{x}_{cur}}$, from the new mean, $\,\overline{{x}_{new}}$ (including the new observation), is statistically significant or not. A key factor is the choice of the cut-off length to start the sequencing that was varied between 5 and 21 years for this 38-year period of study. The regimes presented in Table 1 are found to be stable at 95% confidence interval in the range of variation of cut-off length 5–21. In the second step, when $\,\overline{{x}_{new}}$ is significantly different from $\overline{{x}_{cur}}$, a second criterion, based on a quantity called the ‘Regime Shift Index’ (RSI) is invoked. RSI represents the cumulative sum of normalized anomalies over the current period of analysis (see Rodionov^45^ for the exact equation and its explanation). A regime shift is detected when the new regime mean shows an upward (downward) shift and RSI is negative (positive) and t_cur_ is declared as the change point by the algorithm^46^.

The Changepoint analysis in R^48^ tests for sequential changes in the mean by testing for the null hypothesis (H_0_) that corresponds to no changepoint using a likelihood based framework. The test statistic is constructed using the Maximum Log Likelihood value for the change point. If this Maximum Log Likelihood value is higher than a threshold value then the test rejects the hypothesis.

The Changepoint detection in Matlab^49,50^ involves choosing a point in a timeseries dividing the series into two sections. The total residual error for each section is calculated using the difference between series points and the empirical mean (and/or variance). The changepoint is decided when the total residual error is at a minimum.

The Markov Regime Shift Models^51^ allows for detecting multiple states in a time-series based on estimation of Maximum Log Likelihood^77^. Since the states are unknown, this method involves estimating the Maximum Log Likelihood as a weighted average of the state’s probability distributions. The probabilities of each state are determined by filtered probabilities^78,79^ that use available information of each state based on arrival of new information. A 2-state model was used in this study to detect the regime-shift of the WCR formation.

## Data Availability

The datasets generated during and/or analyzed during the current study are available from the corresponding author on reasonable request.
